# Insecticidal potential of *Cedrus libani* tar in eco-friendly control of cat flea, *Ctenocephalides felis*, from different populations in Türkiye

**DOI:** 10.1016/j.heliyon.2024.e39958

**Published:** 2024-10-30

**Authors:** Ozge Tufan-Cetin, Huseyin Cetin

**Affiliations:** a Department of Environmental Protection Technology, Vocational School of Technical Sciences, Akdeniz University, Antalya, 07070, Türkiye; b Department of Biology, Faculty of Science, Akdeniz University, Antalya, 07070, Türkiye

**Keywords:** Cat flea, *Cedrus libani*, *Ctenocephalides*, Insecticide, Tar

## Abstract

**Purpose:**

The purpose of this research is to evaluate the insecticidal efficacy of cedar (*Cedrus libani* A. Rich.) tar against adults of the cat flea *Ctenocephalides felis* Bouché, a significant ectoparasite affecting both domestic and stray animals.

**Methods:**

Tar was obtained through traditional pyrolytic decomposition of cedar wood in the Elmali district of Antalya, Türkiye. The volatile compounds in the tar were characterized using gas chromatography-mass spectrometry (GC-MS). The tar was tested at various concentrations-100 % (pure tar), 50 % tar, 25 % tar, and 10 % tar. Its efficacy was compared to a 0.5 % concentration of fipronil. Fleas were collected from six locations and exposed to treated filter papers for 1 h. Mortality was assessed after 24 h, and statistical analyses, including one-way ANOVA and probit analysis, were performed to determine LC_50_ and LC_90_ values.

**Results:**

The major components of *Ced. libani* tar identified were β-himachalene (29.16 %) and α-atlantone (28.70 %). The effectiveness of tar was concentration-dependent, with higher concentrations showing flea mortality rates comparable to fipronil. In Kepez-Teomanpaşa, LC_50_ and LC_90_ values were 8.52 % tar and 20.24 % tar respectively, indicating high sensitivity, whereas in Konyaaltı-Pınarbası, LC_50_ and LC_90_ values were 19.48 % tar and 46.91 % tar, suggesting reduced susceptibility. The highest concentration (pure tar) resulted in 100 % mortality across all locations, similar to fipronil.

**Conclusion:**

*Ced. libani* tar demonstrates significant potential as an eco-friendly alternative to chemical insecticides for controlling flea infestations, with its efficacy varying by region. The findings highlight the need for considering regional differences in susceptibility when developing pest control strategies.

## Highlights

• Cedar tar (*Cedrus libani*) is as effective as fipronil against cat fleas.
• Key insecticidal compounds in cedar tar: β-himachalene and α-atlantone.
• Cedar tar efficacy varies by region, suggesting localized pest control needs.

## 1 Introduction

Many organisms defined as pests in our living areas can be vectors for various pathogens, negatively affecting animal and human health and causing damage to our living spaces and stored foods. This ultimately impacts our quality of life and leads to significant financial losses annually. Fleas, an important group among these insects, specifically those belonging to the genus *Ctenocephalides* Stiles & Collins, 1930 (Siphonaptera, Pulicidae), are parasitic insects that commonly affect humans, domestic animals, and stray animals [1,2]. There are approximately 2500 different flea species worldwide, exhibiting a broad geographic distribution and typically living as parasites on specific host species [3,4]. The two most notable species within this genus are *Ctenocephalides felis* Bouché, 1835 (cat flea) and *C. canis* Curtis, 1826 (dog flea), which are commonly found on domestic pets, stray (free-roaming dogs and cats), introduced species (such as rats and foxes), and even wild animals [5]. These fleas are blood-feeding ectoparasites, known for their jumping abilities and specialized mouthparts adapted for piercing the skin and feeding on the blood of their hosts.

The high population of stray animals, especially cats and dogs, in rapidly urbanizing areas of the world results in increased interactions between fleas and humans. Cat and dog fleas can transmit diseases to humans, including flea-borne spotted fever caused by *Rickettsia felis* and cat scratch disease caused by *Bartonella* spp [6,7]. In recent years, there has been a significant rise in complaints about pests, particularly fleas and ticks, affecting stray cats and dogs, including in Türkiye. The lack of regular access to veterinary care and preventive measures for stray animals may be the main reason for the widespread and seemingly unstoppable proliferation of pests such as ticks and fleas globally and in Türkiye.

*Cedrus libani* A. Rich. (Pinaceae), commonly known as the Lebanon cedar and native to the eastern Mediterranean region, is renowned for its traditional use in producing a wood extract, locally called "katran,” especially in southern Türkiye. This extract, used for protecting wooden structures and for medicinal purposes in humans and animals, is typically obtained through a traditional method of pyrolytic decomposition by residents, particularly in the Elmali district of Antalya, Türkiye [8]. The extraction process leverages the unique properties of cedar wood, yielding a fluid substance with a distinctive pungent scent and dark brown to black coloration. Tar has been applied to the skin of domestic animals like goats, sheep, and dogs to protect them against pests such as fleas, lice, flies, and ticks [9]. Additionally, it is added to the drinking water of animals, providing protection for several weeks. The local use of cedar tar in treating ulcer-like wounds and its known antimicrobial properties also suggest potential applications in wound healing and infection prevention. The complex composition of *Ced. libani* tar, influenced by factors such as the age of the tree and extraction technique, contributes to its wide range of beneficial properties [10].

The aim of this research is to evaluate the insecticidal efficacy of *Ced. libani* tar against the adult cat flea, *C. felis*, a significant ectoparasite impacting both domestic and stray animal populations. This assessment is based on the traditional uses of *Ced. libani* tar and seeks to understand its potential as an alternative in combating fleas. Our research investigates the effects of this natural tar at various concentrations on fleas, exploring the possibility of a safer and more environmentally friendly method of pest control for both humans and animals. Thus, we aim to determine whether *Ced*. *libani* tar offers a sustainable and eco-sensitive solution as an alternative to current chemical products used in flea management.

## 2 Material and methods

### 2.1 Tar extraction process

The acquisition of tar involved the utilization of cedar wood (*Ced. libani*) through the traditional pyrolytic decomposition method, practiced by residents residing in Elmali, a district within Antalya, Türkiye. The extraction details are elaborated on in the study by Kurt et al. [8]. According to this study, traditional tar production in the Taurus Mountains is a longstanding distillation process that begins with the excavation of two primary pits: a larger combustion chamber and a smaller collection chamber. The combustion chamber measures approximately 1.0 m in width, 2.0 m in length, and 1.5 m in depth, and it is plastered with mud and clay to prevent leakage and loss of extracts. Positioned just 30 cm below the combustion chamber, the collection chamber houses a receptacle to gather the extracts.

Small wood pieces, known as "çıra," ranging from 10 to 25 cm in length and 2–3 cm in thickness, are densely packed into the combustion chamber. These pieces are strategically arranged to moderate and control the burning process. The combustion chamber is ignited via an opening at the top, which is specifically left open for this purpose.

Once ignited, the temperatures generally exceed 300 °C. Throughout the combustion, controlled aeration is maintained by making small holes in the cover layers, which limits oxygen contact with the fuel and ensures a slow burn. This controlled combustion process, which is both intense and precise, lasts approximately 10–20 min. The tar then flows from the combustion chamber through a discharge channel into the collection chamber. During this process, the flow rate and quality of the tar can vary, influenced by the combustion temperature and air adjustments.

This meticulous and regulated procedure is essential for enhancing the quality and efficiency of traditional tar production. Careful management of temperature and air during the combustion process is crucial to ensuring the production of high-quality tar.

Following the extraction process, the obtained tar was securely stored in a refrigerator at +4 °C until needed.

### 2.2 Analysis of tar composition

The tar's volatile compounds were characterized through gas chromatography coupled with mass spectrometry (GC-MSD), utilizing a suite of sophisticated instrumentation. This ensemble included the Thermo Scientific TRACE 1300 for gas chromatography, paired with the ISQ 7000 mass spectrometry detector and augmented by the RSH Triplus autosampler, all provided by Thermo Fisher Scientific Inc., based in Waltham, MA, USA. The focus was on pinpointing the terpenoid compounds encapsulated in the tar. For the chromatographic analysis, the Xcalibur software, version 4.2.47, was utilized. Effective chromatographic separation was facilitated by the TRACE TR-5MS column, which features a composition of 5 % phenyl and 95 % dimethylpolysiloxane and is measured at 30 m in length and 0.25 mm in diameter, with a film thickness of 0.25 μm, ensuring precision in the analytical process, all sourced from Thermo Fisher Scientific Inc.

The tar sample underwent analysis by being diluted in a 1:500 ratio with acetone before its introduction into a 2 ml vial for injection into the GC-MSD system. This comprehensive analytical procedure lasted 65 min. Key parameters included setting the inlet temperature at 250 °C, determining an injection volume of 2 μl, and establishing a split ratio of 1:20. Helium was utilized as the carrier gas, maintaining a consistent flow rate of 1.5 ml/min. The temperature profile within the oven began at 30 °C, held for the initial 5 min, then increased incrementally by 5 °C each minute until it reached 280 °C, where it was sustained for an additional 10 min. Concurrently, the detector temperature was kept steady at 230 °C. In its operational phase, the mass spectrometer was set to scan from 40 to 550 Da, enabling the detection of a broad range of mass units.

Compound identification involved comparing spectral data against entries in the Wiley and NIST databases. During this process, a specific set comprising basic, molecular, and qualifying ions was selected for every compound recognized, facilitating their accurate identification.

### 2.3 Flea collection and identification

Adult cat fleas were collected from two localities in three central districts of Antalya province (Konyaaltı, Kepez, and Muratpasa). Located on the coast of Antalya, Konyaaltı is a district with a thriving tourism industry, higher socioeconomic status among residents, and a larger population of owned pets. Muratpasa, another central district, is known for its rich cultural and historical landmarks and also has a strong tourism appeal. In contrast, Kepez has more rural characteristics, featuring scattered settlements, and is thought to have a higher population of stray animals [11]. Fleas were collected using hand vacuums from the walls of basements where stray kittens had previously been found and then transferred into 500 ml plastic tubes. Within 1 h, the fleas were transported to the laboratory. Identification of the fleas was carried out using the taxonomic keys provided by the Centers for Disease Control and Prevention and Mathison and Pritt [12] with the aid of a stereomicroscope.

### 2.4 In vitro insecticidal activity of the tar of ced. Libani against cat flea adults

**Fig. 1:**
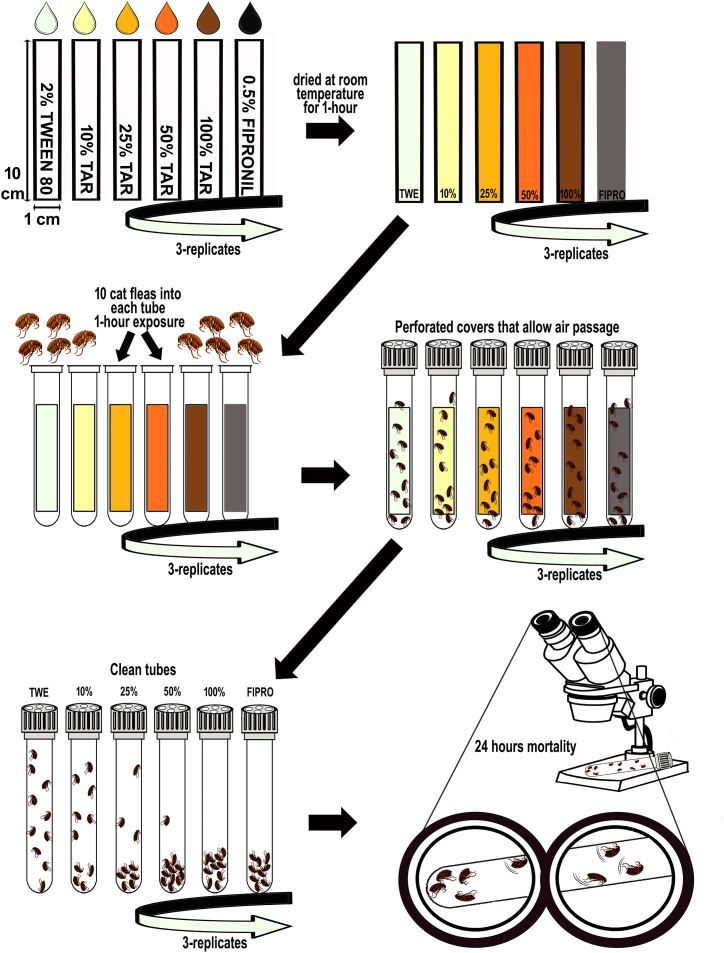
Schematic representation of the in vitro testing procedure for evaluating the insecticidal activity of *Cedrus libani* tar against adult cat fleas.

For the toxicity tests, four concentrations were utilized: pure tar (100 %), 50 % tar, 25 % tar, and 10 % tar dilutions in Tween 80 (2 % v/v) (CAS: 9005-65-6) and distilled water. Filter papers (10 cm^2^) impregnated with 0.25 ml of each concentration were air-dried for 1 h. The tubes have caps with holes that allow air passage. These dried papers were then placed in glass tubes, each containing 10 unfed adult cat fleas (mixed gender), for a 1-h exposure. Although the tar-exposed filter papers were dried in the open for 1 h and the test tubes had holes, the exposure time of fleas to tar was limited to 1 h (Fig. 1). The primary reason for this limitation is that prolonged exposure could lead to fumigant toxicity, potentially increasing the mortality rate. Subsequently, fleas were transferred to clean and empty tubes. Each concentration was tested in triplicate. A Tween 80 (2 % v/v) solution served as the negative control, and a 0.5 % w/v solution of fipronil (CAS: 120068-37-3) was used as the positive control. After 24 h, the number of live adult fleas for each concentration was determined using a stereoscopic microscope, considering fleas that were immobile or weakly moving as deceased. All toxicity tests were conducted at a temperature of 24 ± 2 °C, 40 ± 10 % relative humidity, and a photoperiod consisting of 12:12 h light:dark.

### 2.5 Statistical analysis

We conducted a comprehensive statistical analysis using SPSS software (Version 23.0, SPSS Inc. Chicago, IL, USA) on the collected data. Subsequently, we performed analysis of variance (one-way ANOVA) on the percentage means, followed by Duncan's multiple range test (p ≤ 0.05) to compare the means. Graphs displaying lettering that denotes statistical differences among the data were included in the results section. For the adult mortality data, we applied probit analysis to determine the lethal concentration 50 (LC_50_) rates and lethal concentration 90 (LC_90_) rates, along with their respective confidence intervals.

## 3 Results

Table 1 provides the chemical composition of *Ced. libani* tar, focusing on the major components that constitute more than 1 % of the total composition. Each component is characterized by its specific retention time in minutes, its chemical name (substance), and the percentage of the overall composition that it represents. Notably, the table highlights the prominence of certain compounds within the tar. For instance, β-Himachalene and α-Atlantone emerge as significant constituents, comprising 29.16 % and 28.70 % of the tar's composition, respectively (Table 1).

**Table 1:** Major chemical components in *Ced. libani* tar (Composition Rate >1 %).

No	NIST Library similarity (%)	Retention Time (min)	Substances	Composition Rate (%)
1	97.2	38.27	β-Himachalene	29.16
2	97.4	47.34	α-Atlantone	28.70
3	81.4	45.22	ar-Turmerone	8.82
4	96.8	37.08	Longifolene-(V4)	6.66
5	94.5	35.35	α-Himachalene	5.28
6	90.1	43.62	α-Bisabolol	1.94
7	92.6	32.82	Glcycl-L-proline	1.47
8	80.9	44.10	Veridiflorol	1.38
9	74.2	37.24	1,2,3,4,4a,7-Hexahydro-1,6-dimethyl-4-(1-methylethyl)-naphthalene	1.19

**Fig. 2:**
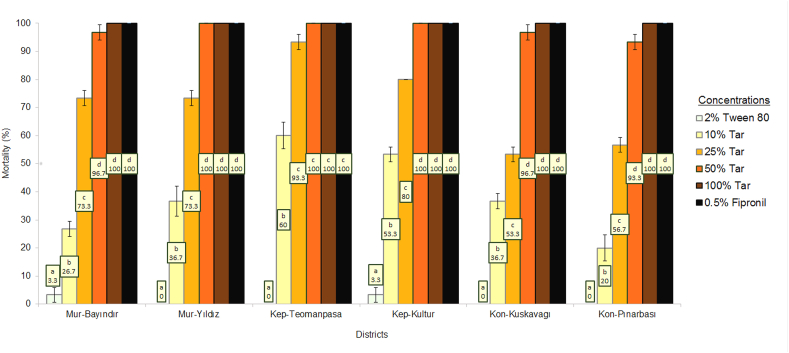
Insecticidal effect of *Cedrus libani* tar on cat fleas (Mean% Mortality ± Standard Error). Percent mortality averages were statistically compared for each location using Duncan's multiple range test (p ≤ 0.05). If the lower-case letters are the same, there is no statistical difference.

*Cedrus libani* tar exhibited potent insecticidal activity at the highest concentrations. Complete mortality (100 %) was observed with both 100 % tar and 0.5 % fipronil across all locations, confirming the tar's efficacy at full strength is comparable to that of fipronil (Fig. 2). However, a decline in flea mortality was noted as the concentration of tar decreased. At 50 % tar concentration, the efficacy was slightly reduced in some instances yet remained high. A more pronounced reduction was observed at 25 % tar concentration, with efficacy ranging from 50 % to 100 % mortality, indicating variability in response across different populations. At 10 % tar concentration, the effectiveness of tar substantially declined, with mortality rates falling between 20 % and 70 %.

The LC_50_ value in Muratpasa-Bayındır was 15.58 % tar, suggesting a moderate response to the tar. In Muratpasa-Yıldız, the LC_50_ was slightly lower at 13.58 % tar, indicating higher flea susceptibility. Kepez-Teomanpasa showed an even lower LC_50_ of 8.52 % tar, reflecting greater effectiveness. The LC_50_ values in Kepez-Kultur and Konyaaltı-Kuskavagı were 10.05 % tar and 16.06 % tar, respectively, while Konyaaltı-Pınarbası recorded the highest LC_50_ at 19.48 % tar, showing reduced susceptibility (Table 2).

**Table 2:** Comparative efficacy of *Cedrus libani* tar in cat flea control: LC_50_ and LC_90_ values across different locations.

Locations	LC_50_ (Limits) as %tar	LC_90_ (Limits) as %tar	Chi-square (χ^2^)	*P*-value
Muratpasa-Bayındır	15.58 (13.50–17.67)	36.60 (31.27–45.16)	0.934	0.627
Muratpasa-Yıldız	13.58 (11.10–15.95)	32.93 (27.13–43.84)	7.903	0.019
Kepez-Teomanpasa	8.525 (6.65–10.04)	20.24 (17.35–25.30)	0.72	0.698
Kepez-Kultur	10.05 (7.91–11.94)	28.70 (24.15–36.45)	7.231	0.027
Konyaaltı-Kuskavagı	16.06 (11.57–20.51)	46.80 (34.83–78.14)	22.047	0.000
Konyaaltı-Pınarbası	19.48 (16.56–22.57)	46.91 (38.83–61.11)	4.571	0.102

Regarding the LC_90_ values, which reflect the concentration needed to kill 90 % of adult fleas, there was notable variation. Muratpasa-Bayındır had an LC_90_ of 36.60 % tar, while Muratpasa-Yıldız had 32.93 % tar. Kepez-Teomanpasa's LC_90_ was significantly lower at 20.24 % tar, indicating higher tar efficacy. Kepez-Kultur showed an LC_90_ of 28.70 % tar. Higher LC_90_ values were observed in Konyaaltı-Kuskavagı and Konyaaltı-Pınarbası, at 46.80 % tar and 46.91 % tar respectively, suggesting lower effectiveness in these areas (Table 2).

Kepez demonstrates the highest susceptibility of fleas to *Ced. libani* tar (as shown by lower LC_50_ and LC_90_ values), suggesting that the tar is most effective in this area. Konyaaltı shows the least susceptibility, indicating that the tar is less effective there. Muratpasa falls in between, with moderate effectiveness (Table 2).

## 4 Discussion

In this study, *Ced. libani* tar demonstrated strong insecticidal activity at higher concentrations, with 100 % mortality observed at full strength, comparable to fipronil. However, as the tar concentration decreased, flea mortality rates also declined, with a significant drop in effectiveness at 10 % tar concentration, where mortality ranged from 20 % to 70 %. These findings suggest a concentration-dependent response to *Ced. libani* tar, with reduced concentrations leading to decreased flea mortality. It is hypothesized that this variability in mortality response may be attributed to the age diversity of the fleas collected from the field, as variations in age among individuals could contribute to differing levels of susceptibility. This highlights the robustness of fipronil as a control agent, which consistently achieved complete control across all tested populations.

Fleas were collected from the basements of buildings where kitten litters are sheltered until they are capable of independence. Our research findings indicate that fleas exposed to *Ced. libani* tar demonstrate regional variations in sensitivity levels. Specifically, fleas from Kepez and Muratpasa were found to be more sensitive to the tar compared to those from Konyaaltı. This difference in sensitivity may be attributed to the use of antiparasitic drugs at various rates among these regions. It was observed that the high socioeconomic status of Konyaaltı district [11] increased the probability of district residents to purchase and use antiparasitic drugs for both their owned pets and stray animals. It was suggested that this may contribute to the increased resistance levels observed in the flea population. This is reflected in the higher LC_50_ and LC_90_ values recorded, suggesting that fleas in Konyaaltı are less susceptible to the effects of *Ced. libani* tar. In contrast, the use of antiparasitic drugs in Kepez and Muratpasa is relatively lower, possibly resulting in a flea population that is more sensitive to the tar, as evidenced by their lower LC_50_ and LC_90_ values. Consequently, the usage patterns of antiparasitic drugs in these distinct districts appear to be a crucial determinant in the varying efficacy of *Ced. libani* tar against fleas in these areas.

The acaricidal activity of tar obtained from different tree species has been studied by researchers. For example, Girişgin et al. [13] investigated the effectiveness of juniper tar (*Juniperus* spp.) smoke against *Varroa destructor* Anderson & Trueman, a significant parasite in bee colonies, but they found it to be ineffective. In a recent study conducted by Koc et al. [9] in our research area, the acaricidal effects of *Ced. libani* tar on *Rhipicephalus* ticks, prominent ectoparasites affecting cats and dogs, were documented. The study revealed that a 1 % tar concentration resulted in mortality rates of 77.7 % for the Konyaaltı strain and 82.2 % for the Kepez strain of the brown dog tick. Regarding LC_50_ and LC_90_ values, the Kepez strain was found to be more sensitive than the Konyaaltı strain. Similarly, the cat flea Kepez strain was found to be more susceptible than the Konyaaltı strain in our research. It was hypothesized that the smaller size and thinner chitinous exoskeleton of tick larvae compared to adult fleas may explain why lower concentrations of tar are more effective against them.

In our research, it is noteworthy that a 0.5 % concentration of fipronil demonstrated a high level of lethal effectiveness against fleas across all locations. Furthermore, our literature review did not identify any studies addressing insecticide resistance in fleas within the Antalya region of Türkiye. The findings of our research are significant, showing that both undiluted tar and its 50 % diluted variant exhibited 100 % efficacy in controlling cat fleas, the same level achieved by fipronil, a widely acknowledged ectoparasiticide. This underscores the potential of tar as a viable alternative for ectoparasite management.

Chemical analyses conducted for our research have identified key active sesquiterpenes in *Ced. libani* tar. Variations in the tar's compound profile are influenced by factors such as extraction methodology, source botanical tissue, extraction duration, and ambient environmental temperatures. Notably, himachalol is present in concentrations ranging from 22.5 % to 32.4 %, β-himachalene at approximately 21.17 %, and α-himachalene between 5.9 % and 10.5 % [14]. Singh and Agarwal [15] demonstrated the significant insecticidal effect of Himalayan cedar tree oil (*Ced. deodara* (Roxb. ex D.Don) G.Don) against *Musca domestica* Linnaeus, 1758 and *Callosobruchus analis* Fabricius, 1781, attributing it to himachalol and β-himachalene, which are predominant in the oil. Additionally, Faris et al. [16] found that natural cis-himachalol from *Ced. atlantica* (Endl.) Manetti ex Carrière, Traité Gén. Conif. essential oil exhibits activity against smooth muscles and various neurotransmitters, suggesting its role in insect neurotoxicity. Correspondingly, it has been observed that himachalenes and atlantones-another group of oxygenated terpenes like himachalenes-constitute over 63 % of cedar tar's profile, indicating potential efficacy for flea management. Complementary field studies have corroborated the efficacy of both undiluted and 50 % diluted tar, achieving mortality rates in cat fleas comparable to those by fipronil, an ectoparasiticide standard. The synergy of laboratory and field data accentuates the potential of *Ced. libani* tar as an effective ectoparasite management agent, warranting its consideration in integrated pest management protocols.

Numerous researchers have investigated the insecticidal effects of extracts and essential oils derived from various plant parts on different life stages of fleas belonging to the genus *Ctenocephalides* [17]. For instance, a study focusing on the larvae of the flea species *C. felis* and *Xenopsylla brasiliensis* Baker, 1904, reported that a 50 % neem (*Azadirachta indica* A. Juss) seed kernel extract demonstrated insecticidal effects on fourth-instar larvae over various exposure times, indicating the extract's potential as a larvicidal agent. After 24 h of exposure, mortality rates were recorded at 95.3 % for *C. felis* and 94.7 % for *X. brasiliensis* [18]. Additionally, a one-time mist-sprayed application of azadirachtin, the primary component of the *A. indica* seed extract, in doses ranging from 1000 to 2400 ppm on dogs, achieved a 93 %–53 % reduction in *C. felis* populations, with effects persisting for 19 days [19]. Lambert et al. [20] explored the efficacy of *Syzygium aromaticum* (L.) Merr. & L.M. Perry essential oil (SAEO) and its component eugenol (EG) against adult fleas and their eggs in *C. felis*. The research demonstrated that both SAEO and EG effectively killed adult fleas, with EG showing a higher pulicidal efficiency at lower concentrations. The study also found that SAEO and EG inhibited the development of flea eggs in adults.

Extracts of ethyl acetate, n-hexane, and methanol, along with essential oils from the leaves and mature fruits of *Schinus molle* L., an endemic tree species in Brazil, were tested in vitro against adults and eggs of cat fleas (*C. felis*). At a dosage of 800 μg/cm^2^, the essential oils from both the fruit and leaves achieved 100 % efficacy. Among the extracts, only the n-hexane extract demonstrated strong activity at this dosage [21]. Furthermore, the insecticidal effects of lemon fruit (*Citrus limon* L.) on adult dog fleas (*C. canis*) were explored by Garnace et al. [22]. They compared two concentrations of lemon fruit extract (50 % and 100 %) against commercial dog flea shampoo and water. The study revealed that the 100 % lemon extract concentration and the commercial shampoo exhibited the highest mortality rates, surpassing those achieved by the 50 % lemon extract concentration, with water showing the lowest mortality.

The insecticidal effects of essential oils derived from the leaves of five plants and the stem of one plant on the adult, larval, and egg stages of the cat flea (*C. felis*) were investigated by Dos Santos et al. [23]. They found that the essential oil from *Ocimum gratissimum* L. was the most effective across all life stages of the fleas. In a subsequent study, Oliveira et al. [24] examined the efficacy of *Oc. gratissimum* essential oil and its principal component, eugenol, specifically against adult cat fleas. Their findings revealed that eugenol achieved 100 % mortality at a concentration of 1200 μg/ml, whereas the essential oil of *Oc. gratissimum* required a higher concentration of 2500 μg/ml to attain equivalent efficacy.

The insecticidal activity of cassia (*Cinnamomum cassia* (L.) J. Presl), thyme (*Thymus vulgaris* L.), and oregano (*Origanum vulgare* L.) volatile oils against adult stages of cat fleas studied by Conceição et al. [25]. They reported that for adult fleas, the oregano volatile oil showed the best efficacy, with an LC_50_ value of 33.5 μg/cm^2^ at 24 h and 21.8 μg/cm^2^ at 48 h. Thyme volatile oil also demonstrated significant adulticidal activity, with LC_50_ values of 64.5 μg/cm^2^ at 24 h and 44.7 μg/cm^2^ at 48 h. *Cassia* volatile oil, while effective, required higher concentrations to achieve similar mortality rates, with LC_50_ values of 74.7 μg/cm^2^ at 24 h and 64.3 μg/cm^2^ at 48 h. Freitas et al. [26] investigated the insecticidal activity of *Illicium verum* Hook. and *Pelargonium graveolens* L′ Hér. essential oils against adult fleas and their immature stages. Their findings indicated that the essential oil of *I. verum* exhibited insecticidal activity for about 18 days, whereas the activity of *P. graveolens* essential oil persisted for 13 days.

It has been observed that local people occasionally add *Cedrus* tar to the drinking water of domestic animals such as goats, dogs, and cats. According to local accounts, this practice not only improves the animals' health but also reduces the incidence of ecto- and endoparasites, presumably due to the bioactive components present in the tar. However, to substantiate these claims, there is a need for new controlled experiments involving these animal species. In a related context, Banuls et al. [27] reported on the efficacy of a dietary plant extract formulation (Bioticks®) that includes lemon balm, lemongrass, fenugreek, rosemary, thyme, and wormwood extracts in reducing flea populations in naturally infested cats with an indoor-outdoor lifestyle. Their findings indicate that this plant-based food supplement significantly diminished flea populations over five months. The efficacy rates increased progressively, reaching 77 % by the end of the study period when compared to a placebo group.

## 5 Conclusion

Our research indicates that tar obtained from *Ced. libani* demonstrates substantial in vitro effectiveness against cat fleas, positioning it as a viable candidate for further exploration in developing eco-friendly and non-toxic flea control solutions. Although plant-derived products, notably essential oils and extracts, may be perceived as safe for animals like cats and dogs, research by Genovese et al. [28] has highlighted potential adverse reactions. Specifically, cats exhibited agitation and hypersalivation, whereas dogs showed signs of lethargy and vomiting when exposed to plant-based flea preventatives containing essential oils, even when these products were applied as per the manufacturer's instructions.

Given tar's complex composition with multiple bioactive components, it is critical to understand their interactions-whether synergistic or antagonistic-as these can significantly affect its effectiveness and safety as a flea treatment. Moreover, identifying the mechanisms through which tar impacts fleas, such as targeting acetylcholinesterase or GABA receptors, is crucial for determining its action mode.

## CRediT authorship contribution statement

**Ozge Tufan-Cetin:** Writing – review & editing, Writing – original draft, Visualization, Software, Methodology, Formal analysis, Data curation. **Huseyin Cetin:** Writing – review & editing, Writing – original draft, Visualization, Software, Methodology, Investigation, Formal analysis, Data curation, Conceptualization.

## Availability of data and material

Data is provided within the manuscript.

## Ethical statements

Ethics approval was not required for this study as no research was conducted on vertebrate animals.

## Funding

The authors did not receive support from any organization for the submitted research.

## Declaration of competing interest

The authors declare that they have no known competing financial interests or personal relationships that could have appeared to influence the work reported in this paper.

## References

[1] Coskun G., Cetin H. (2018). A research about flea (Siphonaptera: Pulicidae) infestation on domestic cats and dogs in winter months, from Antalya, Turkey. Turk. Parazitoloji Derg..

[2] Yevstafieva V., Horb K., Melnychuk V., Bakhur T., Feshchenko D. (2020). Ectoparasites Ctenocephalides (Siphonaptera, Pulicidae) in the composition of mixed infestations in domestic dogs from Poltava, Ukraine. Folia Vet..

[3] Dobler G., Pfeffer M. (2011). Fleas as parasites of the family Canidae. Parasites Vectors.

[4] Durden L.A., Hinkle N.C. (2019). Medical and Veterinary Entomology.

[5] Clark N.J., Seddon J.M., Šlapeta J., Wells K. (2018). Parasite spread at the domestic animal-wildlife interface: anthropogenic habitat use, phylogeny and body mass drive risk of cat and dog flea (*Ctenocephalides* spp.) infestation in wild mammals. Parasites Vectors.

[6] Bitam I., Dittmar K., Parola P., Whiting M.F., Raoult D. (2010). Fleas and flea-borne diseases. Int. J. Infect. Dis..

[7] El Hamzaoui B., Zurita A., Cutillas C., Parola P. (2020). Fleas and flea-borne diseases of north africa. Acta Trop..

[8] Kurt Y., Kaçar S.M., Işık K. (2008). Traditional tar production from *Cedrus libani* a Rich. the Taurus mountains in southern Turkey. Econ. Bot..

[9] Koc S., Gultekin Z.N., Kahraman S., Cengiz A., Polat B., Caliskan C., Yildirim T., Tufan-Cetin O., Cetin H. (2023). Exploring the larvicidal and repellent potential of Taurus cedar (*Cedrus libani*) tar against the brown dog tick (*Rhipicephalus sanguineus* sensu lato). Molecules.

[10] Avcı A., Özen R. (2016). Use of "Black Doctor: tar" for the treatment of animal diseases as part of the veterinary medical folklore of Antalya province. Fırat Üniversitesi Sağlık Bilimleri Veteriner Dergisi.

[11] Acar S., Karagöz T., Meydan M.C., Cinoğlu D.Ş., Kaygısız G., Işık M. (2022).

[12] Mathison B.A., Pritt B.S. (2014). Laboratory identification of arthropod ectoparasites. Clin. Microbiol. Rev..

[13] Girişgin O., Çakmak İ., Çakmak S.S., Aydın L. (2007). Is juniper tar smoke effective against Varroa? U. Arı. D.-. Uludağ Bee Journal.

[14] Kurt Y., Işık K. (2012). Comparison of tar produced by traditional and laboratory methods. Stud. Ethno-Med..

[15] Singh D., Agarwal S.K. (1988). Himachalol and β-himachalene: insecticidal principles of Himalayan cedarwood oil. J. Chem. Ecol..

[16] Faris A., Edder Y., Louchachha I., Lahcen I.A., Azzaoui K., Hammouti B., Merzouki M., Challioui A., Boualy B., Karim A., Hanbali G., Jodeh S. (2023). From himachalenes to trans-himachalol: Unveiling bioactivity through hemisynthesis and molecular docking analysis. Sci. Rep..

[17] Soares E.F.M.S., Carlos D.F.L.P., Epifanio N.M.M., Coumendouros K., Cid Y.P., Chaves D.S.A. (2023). Insecticidal activity of essential oil of *Cannabis sativa* against the immature and adult stages of *Ctenocephalides felis felis*. Braz. J. Vet. Parasitol..

[18] Kilonzo B.S. (1991). Larvicidal effects of neem, *Azadirachta indica* on fleas in Tanzania. Int. J. Trop. Insect Sci..

[19] Guerrini V.H., Kriticos C.M. (1998). Effects of azadirachtin on *Ctenocephalides felis* in the dog and the cat. Vet. Parasitol..

[20] Lambert M.M., Campos D.R., Borges D.A., de Avelar B.R., Ferreira T.P., Cid Y.P., Boylan F., Scott F.B., de Almeida Chaves D.S. (2020). Activity of *Syzygium aromaticum* essential oil and its main constituent eugenol in the inhibition of the development of *Ctenocephalides felis felis* and the control of adults. Vet. Parasitol..

[21] Batista L.C.S.O., Cid Y.P., Almeida A.P., Prudêncio E.R., Riger C.J., Souza M.A.A., Chaves D.S.A. (2016). In vitro efficacy of essential oils and extracts of *Schinus molle* L. against *Ctenocephalides felis felis*. Parasitology.

[22] Garnace J.M., Obsequies I.C., Binoya H.L., Tarona L.M., Flores M.B. (2019). Insecticidal effect of *Citrus limon* (lemon fruit) extract on adult *Ctenocephalides canis* (dog flea). Cebu Doctors' University Thesis/Dissertations.

[23] Dos Santos J.V.B., de Almeida Chaves D.S., de Souza M.A.A., Riger C.J., Lambert M.M., Campos D.R., Moreira L.O., Dos Santos Siqueira R.C., de Paulo Osorio R., Boylan F., Correia T.R., Cid Y.P. (2020). In vitro activity of essential oils against adult and immature stages of *Ctenocephalides felis felis*. Parasitology.

[24] Oliveira L.M., de Almeida Chaves D.S., Raquel de Jesus I.L., Miranda F.R., Ferreira T.P., Nunes e Silva C., de Souza Alves N., Alves M.C.C., Avelar B.R., Scott F.B., Campos D.R., Cid Y.P. (2022). *Ocimum gratissimum* essential oil and eugenol against *Ctenocephalides felis felis* and *Rhipicephalus sanguineus*: in vitro activity and residual efficacy of a eugenol-based spray formulation. Vet. Parasitol..

[25] Conceição C.L., Morais L.A.S., Campos D.R., Chaves J.K.O., Santos G.C.M., Cid Y.P., Sousa M.A.A., Scott F.B., Chaves D.S.A., Coumendouros K. (2020). Evaluation of insecticidal activity of thyme, oregano, and cassia volatile oils on cat flea. Revista Brasileira de Farmacognosia.

[26] Freitas J.P., Jesus I.L.R., Chaves J.K.O., Gijsen I.S., Campos D.R., Baptista D.P., Ferreira T.P., Alves M.C.C., Coumendouros K., Cid Y.P., Chaves D.S.A. (2021). Efficacy and residual effect of *Illicium verum* (star anise) and *Pelargonium graveolens* (rose geranium) essential oil on cat fleas *Ctenocephalides felis felis*. Braz. J. Vet. Parasitol..

[27] Banuls D., Brun J., Blua J.L., Cadiergues M.C. (2023). A dietary plant extract formulation helps reduce flea populations in cats: a double-blind randomized study. Pharmaceuticals.

[28] Genovese A., McLean M., Khan S. (2012). Adverse reactions from essential oil-containing natural flea products exempted from Environmental Protection Agency regulations in dogs and cats. J. Vet. Emerg. Crit. Care.

